# Large-scale phase retrieval

**DOI:** 10.1038/s41377-021-00616-4

**Published:** 2021-09-03

**Authors:** Gabriel Popescu

**Affiliations:** Quantitative Light Imaging Laboratory, Department of Electrical and Computer Engineering, Beckman Institute for Advanced Science and Technology, University of Illinois at Urbana-Champaign, Urbana, IL 61801 USA

**Keywords:** Imaging and sensing, Displays

The *coherent* interaction between an electromagnetic field and a 3D weakly scattering medium results in a simple Fourier transform relationship between the object’s structure and the *complex* scattered field^1^. As a result, knowledge about the phase of the scattered field is necessary for solving this inverse problem with a unique solution. However, in applications, such as X-ray crystallography, typically one only has experimental access to the *amplitude* of the diffracted field, which results in ambiguities of the reconstruction. This century-old challenge, known as “the phase problem”^2^ motivated the development of computational algorithms combined with a priori knowledge about the object of interest to limit the solution space. While generally successful, this approach sometimes led to multiple solutions, i.e., different inferred object structures for the same set of data: “Not all the guesses have been successful. This is clear, for example, from the following: Two different structures were predicted for the mineral bixbyite, one by L. Pauling, the other by W. H. Zachariasen. It is not known which, if either, is correct.” (Chapter 7 in ref. ^2^).

In the optical regime, due to the luxury of available coherent sources, virtually perfect lenses, and high-resolution detectors, extracting the phase of the scattered field from intensity measurements has been a solved problem for many decades, using off-axis^3^ or phase-shifting interferometry^4^. In recent years, quantitative phase maps associated with transparent biospecimens have found multiple biomedical applications^5^, in a rising field known as quantitative phase imaging (QPI)^6^. Traditional interferometric systems (e.g., Michaelson, Mach-Zehnder) are typically associated with experimental complexity, temporal noise due to temporal instability, and spatial noise due to laser speckles. However, recent interests in QPI have triggered the development of stable, speckle-free methods that can be used for routine biological investigations^7^.

Despite these *experimental* advances in phase retrieval, *computational* algorithms have continued to develop even for optical domain applications, mainly to afford simple imaging systems and take advantage of the computational power that is now readily available. Of course, this simpler optical approach brings back the convergence problems associated with all computational phase retrieval methods^8^. The study by Chang et al. ^9^ tackles this challenge and provides a solution for high space-bandwidth phase retrieval using deep learning. The authors demonstrated that their technique holds some advantages over existing alternatives: “Extensive simulations and experiments validate that the technique outperforms the existing PR algorithms with as much as 17 dB enhancement on signal-to-noise ratio, and more than one order-of-magnitude increased running efficiency.” At the same time, they acknowledge remaining convergence issues that grant further investigation: “The PNP (plug-and-play) framework has a theoretical guarantee of convergence for most real-domain tasks, such as denoising, deblurring, etc. However, to the best of our knowledge, there is no theoretical proof of PNP’s convergence in the complex domain. Further, there is also no theoretical guarantee of convergence for the alternating projection solver that has been widely used for ~50 years.” The authors’ correct assertion is not a shortcoming of their computational algorithm, but rather, it is rooted in the physics of image formation. The intensity detected in Gabor’s in-line holography (Chang et al. ^9^), or any other interferometry experiments for that matter, simply contains too many unknowns in a single measurement of intensity *I*, namely

1 $$I(x,y) \,=\, \left| {A_1(x,y)e^{i\phi _1(x,y)} \,+\, A_2(x,y)e^{i\phi _2(x,y)}} \right|^2$$

where *A*_1,2_ are the unknown amplitudes and ϕ_1,2_ are the unknown phases of the two interfering fields. Thus, without prior knowledge about the object, a single intensity measurement is simply insufficient for extracting the phase difference ϕ_2_-ϕ_1_ of interest.

It is likely that such fast deep learning-enabled algorithms, like the ones presented by Chang et al. ^9^, will be valuable in combination with optical experiments that can solve the convergence problem. At the same time, today there is an enormous opportunity in the biophotonics field for boosting the resolution^10^ and chemical specificity^11^ of optical systems, as well as extracting accurate medical information from optical imaging data^12–14^.
